# Investigating Effects of Proteasome Inhibitor on Multiple Myeloma Cells Using Confocal Raman Microscopy

**DOI:** 10.3390/s16122133

**Published:** 2016-12-14

**Authors:** Jeon Woong Kang, Surya P. Singh, Freddy T. Nguyen, Niyom Lue, Yongjin Sung, Peter T. C. So, Ramachandra R. Dasari

**Affiliations:** 1 Laser Biomedical Research Center, G. R. Harrison Spectroscopy Laboratory, Massachusetts Institute of Technology, Cambridge, MA 02139, USA; spsingh@mit.edu (S.P.S.); freddytn@mit.edu (F.T.N.); niyom@mit.edu (N.L.); ysung4@uwm.edu (Y.S.); ptso@mit.edu (P.T.C.S.); rrdasari@mit.edu (R.R.D.); 2 Department of Chemical Engineering, Massachusetts Institute of Technology, Cambridge, MA 02139, USA; 3 Department of Mechanical Engineering, Massachusetts Institute of Technology, Cambridge, MA 02139, USA; 4 Department of Biological Engineering, Massachusetts Institute of Technology, Cambridge, MA 02139, USA

**Keywords:** Raman spectroscopy, Raman microscopy, therapeutic response monitoring, cell imaging

## Abstract

Due to its label-free and non-destructive nature, applications of Raman spectroscopic imaging in monitoring therapeutic responses at the cellular level are growing. We have recently developed a high-speed confocal Raman microscopy system to image living biological specimens with high spatial resolution and sensitivity. In the present study, we have applied this system to monitor the effects of Bortezomib, a proteasome inhibitor drug, on multiple myeloma cells. Cluster imaging followed by spectral profiling suggest major differences in the nuclear and cytoplasmic contents of cells due to drug treatment that can be monitored with Raman spectroscopy. Spectra were also acquired from group of cells and feasibility of discrimination among treated and untreated cells using principal component analysis (PCA) was accessed. Findings support the feasibility of Raman technologies as an alternate, novel method for monitoring live cell dynamics with minimal external perturbation.

## 1. Introduction

Cellular homeostasis is maintained by a finely regulated network consisting of ubiquitin-proteasome pathway. It is responsible for the degradation of most regulatory proteins involved in apoptosis, cell growth and division, and DNA repair pathways [1,2]. Unneeded or damaged proteins are tagged by ubiquitin to be directed to the proteasomes and finally degraded to maintain the balance of inhibitory and stimulatory proteins. Disruption of this pathway during cancer development and other diseases lead to cell cycle arrest and cell death [2]. Cancer cells are well-known to have high proteasome activity, making them an ideal target for therapeutic interventions. Based on promising clinical trials, Bortezomib has been extensively explored as a therapeutic strategy to treat multiple myeloma [3]. Its introduction to the treatment of multiple myeloma has been a breakthrough especially in relapsed cases.

Bortezomib, originally named as PS-341, was the first-in-class proteasome inhibitor to be clinically introduced. It is a boron containing molecule that specifically and reversibly inhibits the threonine residue of the 26S proteasome, an enzyme complex that plays a key role in regulating protein degradation. Bortezomib blocks the removal of nonfunctional proteins by inhibiting proteasomes leading to accumulation of abnormal proteins and ultimately cell death [4,5]. In multiple myeloma, the mechanism of action of Bortezomib disrupts cellular signaling adversely influencing the tumor microenvironment and cell adhesion processes [6,7]. Bortezomib also inhibits DNA repair, angiogenesis, and osteoclast activity [8].

Monitoring and measuring the treatment response and effectiveness has been a major growing area of cancer research. Non-invasive tools that can rapidly measure drug response in a quantifiable and label-free manner are highly desirable [9]. Optical spectroscopy offers a promising alternative to existing chemical assays, which provide observations only at a fixed time point, involve biopsies or sample removal and intensive labor. Since its discovery in 1928, Raman scattering has been widely used as an analytical tool for many applications in the laboratory [10]. In-elastically scattered Raman light from the sample, which includes the “fingerprint” vibrational information, can be used for both qualitative and quantitative analysis. Compared to other optical methods, Raman spectroscopy has several advantages for medical diagnostics. It does not require an external label or marker, and is least susceptible by water absorption and therefore readily adaptable for in vivo measurements. As the Raman fingerprint contains rich biological information, variations due to disease or inflammatory processes can be readily observed in the spectral profile [10]. Applications for Raman spectroscopy are rapidly growing, as a tool for disease diagnosis, monitoring disease progression post-treatment, and evaluating treatment effectiveness [11,12].

Confocal microscopy has been widely used to acquire three-dimensional information of biological samples. In confocal microscopy, a pinhole rejects the out of focus light resulting in higher axial resolution than wide-field microscopy. The confocal technique can be combined with reflectance, fluorescence and inelastic scattering measurements such as Raman and Brillouin, providing a three-dimensional mapping of these signals [13,14]. Integrating confocal microscopy with conventional Raman spectroscopy provides exciting new research opportunities, owing to the possibility of acquiring and mapping biologically relevant chemical information along with morphological and structural components with high spatial resolution. Confocal Raman microscopy was first used in cells by Puppels et al. in 1990 [13]. Despite its great promise, the use of this technique has been limited in biological research, in comparison to fluorescence imaging, due to the intrinsically weaker Raman signals. Instead of mapping the Raman signal of the entire cell with high spatial resolution, which can be time-intensive, our laboratory initially identified morphologically relevant cellular features using bright-field microscopy followed by measuring the Raman spectra from specific points of interest [15]. In this approach, Raman basis spectra were collected, correlated to specific cellular features, and used to develop the Raman clinical instruments and algorithms previously reported [16]. This hybrid approach has been highly successful in balancing the needs for high-speed and high-resolution Raman imaging.

With recent advancement in spectrograph and CCD technology, Raman cellular mapping was successfully demonstrated with higher spatial and temporal resolution [17]. However, employing this technique for monitoring intracellular chemical distribution in real-time continues to be a challenge due to the long acquisition time needed to acquire weak Raman signal. To overcome this limitation, the MIT Laser Biomedical Research Center developed a high-speed confocal Raman microscopy system for live cell imaging in 2011 [18]. By upgrading to a more sensitive detector (>95% quantum efficiency at 850 nm) combined with high-throughput optical components with enhanced near infrared (NIR) transmission, Raman scattered light can be more efficiently collected with shorter exposure time. NIR Raman images from live cell can be obtained within four minutes [19,20]. Instead of using XY mechanical stages to move the sample, galvanometer mirrors were incorporated to simultaneously raster scan the laser beam across the sample and collect Raman signals from the sample. This integrated system has been successfully utilized for the diagnosis of malaria by measuring the hemoglobin and hemozoin distributions in the infected red blood cells [18]. The uptake of single walled carbon nanotubes by a live cell was monitored with one-minute temporal resolution over an hour [19]. Three-color imaging using surface enhanced Raman scattering (SERS) nanoparticles was achieved with 27.5 s temporal resolution [20]. Alternative glycemic markers such as glycemic hemoglobin and glycemic albumin were measured with lower than physiological concentrations [21,22]. In this paper, we present our high-speed NIR confocal Raman microscope system and its application for live cell imaging and monitoring real-time cell-drug interaction on multiple myeloma cells (RPMI-8226).

## 2. Materials and Methods

### 2.1. Instrumentation

**Figure 1:**
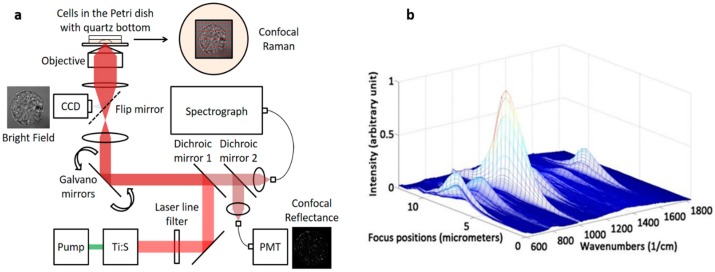
(**a**) Schematic of high-speed confocal Raman microscopy system; and (**b**) axial resolution test by moving a 1-µm diameter polystyrene bead through the focal plane.

A custom-built NIR confocal Raman microscopy system (Figure 1a) with a 785 nm excitation wavelength (Ti:Sapphire laser—3900S, Spectra-Physics, Santa Clara, CA, USA) was developed. The collimated beam was filtered by a band pass filter (BPF, LL01-785-12.5, Semrock, Rochester, NY, USA) and redirected to the dual axes galvanometer mirrors. High-speed XY scanning was performed using galvanometer mirrors (CT-6210, Cambridge Technology, Bedford, MA, USA). A 1.2 NA water immersion objective lens (UPLSAPO60XWIR 60X/1.20, Olympus, Tokyo, Japan) was used to focus the laser light onto the cell and to collect the back-scattered light. A piezo actuator combined with a differential micrometer (DRV517, Thorlabs, Newton, NJ, USA) was used for coarse and fine focusing, respectively, of the sample. A flip mirror was placed after the tube lens to project the sample focal plane onto the video camera with 67× magnification. The back-scattered Raman light from the sample passes through two dichroic mirrors (DM1 and DM2: LPD01-785RU-25, Semrock) and was collected with a multi-mode fiber (M14L01, Thorlabs). Depending on the sample, confocal sampling volume can be easily adjusted by changing the core size of the multi-mode fiber. The collected signal was delivered to the spectrograph (Holospec f/1.8i, Kaiser Optical Systems, Ann Arbor, MI, USA) and detected by a thermoelectric-cooled, back-illuminated and deep depleted CCD (PIXIS: 100BR_eXcelon, Princeton Instruments, Trenton, NJ, USA). The spectrograph covers a spectral range from −34 to 1894 cm^−1^ with 2 cm^−1^/pixel resolution. The rejected Rayleigh light from the second dichroic mirror (DM2) was collected by a single-mode fiber (P1-780A-FC cm^−1^, Thorlabs) and delivered to a photomultiplier tube (PMT, H9656-20, Hamamatsu, Hamamatsu, Japan), which was amplified by a PMT controller (C7169, Hamamatsu, Hamamatsu, Japan). This setup provides co-registration pixel to pixel of the Raman and confocal reflectance images, which were used to optimize the field of view for the Raman mapping. LabView software (National Instruments, Austin, TX, USA) and a data acquisition board (PCI-6251, National Instruments) were used to control the system, acquire the data, and analyze the data. CMOS camera (BCN-B050-U, Mightex, Toronto, ON, Canada) was also used to capture bright field images.

### 2.2. Axial Resolution Test

Prior to cellular imaging, the axial resolution was measured using 1-µm diameter polystyrene beads (07310-15, Polysciences, Inc., Warminster, PA, USA). Polystyrene beads were dispersed in methanol and dried on top of a quartz coverslip (043210-KJ, Alfa Aesar, Haverhill, MA, USA). A series of Raman spectra were collected while the sample was moved in and out of the focal plane using the piezo actuator. The intensity of the polystyrene Raman spectra changes with respect to the sample position and is strongest when in focus (Figure 1b).

### 2.3. Cell Culture

Multiple myeloma cells (RPMI-8226) were purchased from ATCC and maintained in the culture media (RPMI-1640) with 10% fetal bovine serum and 1% antibiotics. In order to maintain cellular viability and minimize the substrate background interference during Raman imaging, the cells were cultured on custom Petri dishes with a bottom quartz cover slip (043210-KJ, Alfa Aesar, Ward Hill, MA, USA) for at least 48 h prior to imaging allowing the cells to adhere to the quartz substrate. Bortezomib (Velcade, Millennium Pharmaceuticals, Cambridge, MA, USA) was introduced into the cell medium at a concentration of 50 nM.

### 2.4. Spectral Acquisition from Single and Group of Cells

From a single multiple myeloma cell, Raman spectra (30 × 30 pixels) were acquired from a 16 µm × 16 µm area with exposure time of 0.5 s/pixel at 20 mW laser power. No visible changes due to laser exposure were observed. The EC_50_ dose for Bortezomib in RPMI-8226 cells when exposed for 48 h was previously reported to be 46 nM [4]. To determine the earliest effects of Bortezomib at this concentration on multiple myeloma, three Raman images of same cell were acquired with one-hour intervals. For each Raman image, the measurement time was approximately 7.5 min. K-means cluster imaging using Raman tools package code was performed to generate Raman images [23]. This method was previously employed in number of studies [24,25]. Four cluster images were generated for each cell at *t* = 0, *t* = 1 and *t* = 2 h after drug treatment. Spectra from each cluster corresponding to an intracellular region were extracted and baseline corrected by fitting a 5th order polynomial function.

Based on the initial observation with single cell, the experiment was repeated on larger groups of cells (~400 cells). To effectively acquire the Raman signal and to avoid the sampling limitation from small confocal volume while keeping the high collection efficiency, we integrated the Raman scattered light from the entire cell by opening the camera shutter while the focused beam scanned the cell. With this method, Raman spectrum of the entire cell can be acquired in 5 s. Two culture dishes were prepared: one was treated with 50 nM of Bortezomib while the other was untreated. Following a four-hour incubation period, single Raman spectrum per cell was acquired for each of the 400 treated cells and the 400 untreated cells. Using a 785 nm laser with 60 mW excitation power, the entire cell was raster scanned in 5 s. By raster scanning the entire cell with the open shutter, a larger number of cells were simultaneously monitored.

### 2.5. Multivariate Analysis

In order to analyze statistical differences among spectra acquired from group of treated and untreated cells, principal component analysis (PCA), was performed. It is one of the most commonly utilized unsupervised approach to extract key variables describing the large variances within a data set [26]. It is mostly used for data overviewing and identifying pattern or outliers. PCA describes data variance by identifying a new set of orthogonal features known as principal components (PCs) or factors [26]. This technique can be used to reconstruct spectra using only the significant principle components (PCs), thus retaining important spectral data while removing background noise. Unprocessed spectra acquired from each of the untreated and treated cells (after removing outliers) were fed into a custom MATLAB based algorithm and scatter plots were generated discriminating between the two groups [26]. Loading plots of factors used for classification were also generated.

## 3. Results and Discussion

### 3.1. Testing Axial Resolution of the System

One major advantage of a custom-built system is its high flexibility. Depending on the sample, the sampling volume can be easily adjusted by changing the configuration of the collection fiber. For example, high-spatial resolution is not necessary to acquire Raman spectra from large number of cells where bulk characterization and statistical averaging are more critical. Collecting the maximum Raman signal with a large-core collection fiber is more important than maintaining a high-spatial resolution. On the other hand, maintaining high-spatial resolution is more critical if the sample has well-defined morphological structure such as cells and microstructures. Without this high-spatial resolution capability, the imaging system would be unable to distinguish the Raman signals coming from different intracellular organelles. The axial resolution was measured using a 50-µm core collection fiber and polystyrene beads (1-µm diameter) deposited on a quartz cover slip. Using an isolated bead, a series of Raman spectra were taken by moving the focal plane in 300 nm increments. In Figure 1b, the changing Raman signal is shown at different focal positions. The axial resolution is determined by the full width half maximum (FWHM) of the strongest Raman peak (1001 cm^−1^) and was measured to be 2.2 µm, which is a value between the maximum confocal resolution (1.1 µm) and cell thickness.

### 3.2. Study on Single Cells

**Figure 2:**
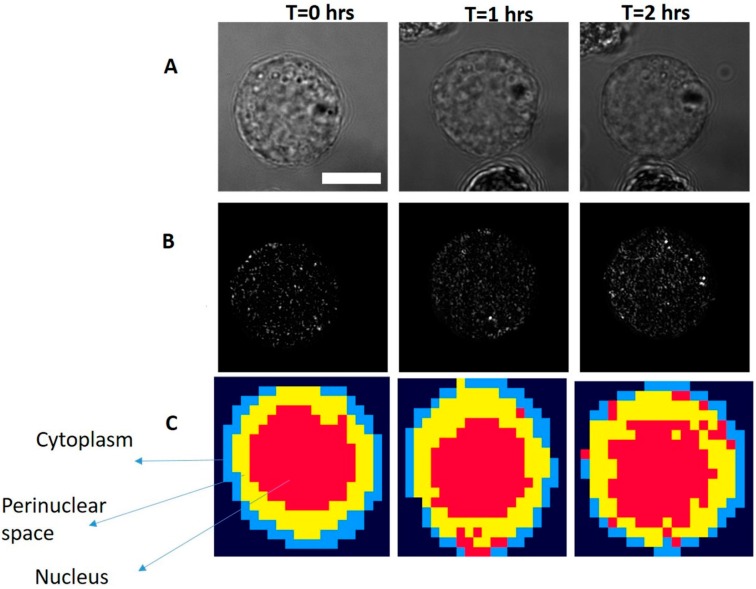
Temporal monitoring of the same RPMI8226 cell after applying 50 nM Bortezomib at time zero (Scale bar: 5 µm): (**A**) bright field image; (**B**) confocal reflectance (binary mask is applied to target cell to eliminate the effect from outside of the cell); and (**C**) Four cluster Raman images. Different intracellular regions are marked.

RPMI-8226 come from the peripheral blood and have the typical morphology of B-lymphocytes. These are characterized by a nearly spherical shape, single large condensed nucleus, perinuclear space and a thin layer of cytoplasm. In Figure 2A, bright field images, confocal reflectance images, and four cluster Raman images are shown. As can be seen from cluster Raman images, different cellular regions such as the thin layer of cytoplasm (blue), perinuclear space (yellow), and nucleus (red) can easily be demarcated. Differentiation between the cellular regions are easily distinguishable in untreated cells (*t* = 0) as compared to Bortezomib-treated cells. With the Bortezomib-treated cells, irregular distribution of the nuclear content can be observed from cluster images. The accumulation of the damaged DNA promotes cellular apoptosis. Disruption in the nuclear content and morphological changes such as cell blebbing are considered hallmarks of apoptosis [27]. As shown in cluster images, cell after two hours of Bortezomib treatment demonstrate morphological changes and the intermixing of cellular regions. Irregular distribution of cytoplasmic content in treated cell suggests changes in the morphological and architectural arrangements of the cellular components leading towards cell death. This correlates with the mechanism of action of proteasome inhibitors on proliferating malignant cells. These cells have defective cell cycle proteins leading to increased accumulations of damaged proteins and enhanced dependency on proteasomal degradation. Bortezomib works by inhibiting the adhesion of multiple myeloma cells to bone marrow stroma, followed by induction of several signaling pathways that impair the DNA-repair pathways. This leads to the down-regulation of anti-apoptotic pathways triggering apoptosis in multiple myeloma cells at concentrations that leave normal cells unaffected. Furthermore, the observed morphological changes at the cytoplasmic envelope closely correspond to the accumulation of poly-ubiquitinated proteins previously reported from immunofluorescence staining experiments [4].

**Figure 3:**
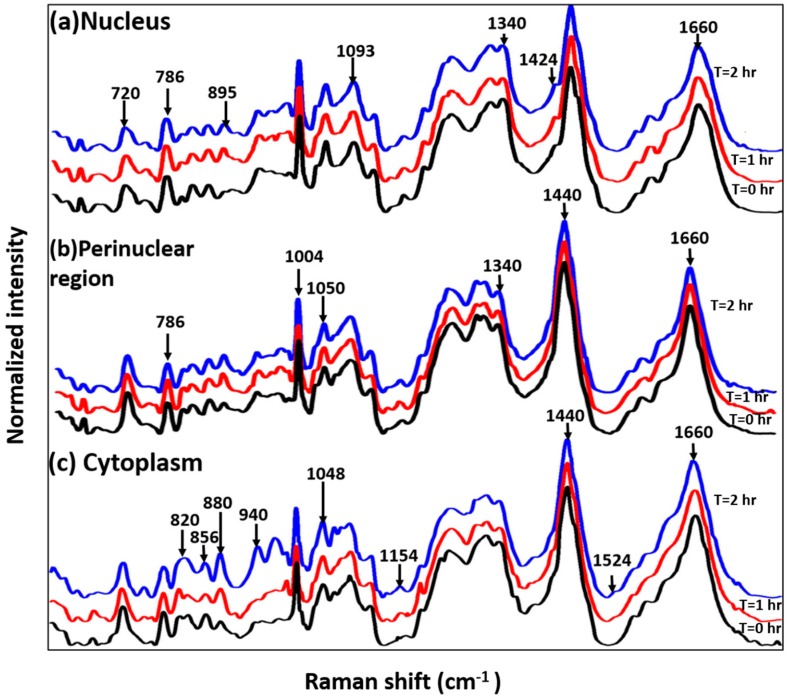
Mean baseline corrected spectra extracted from individual clusters annotated to an intracellular region in cluster Raman image: (**a**) Nucleus; (**b**) Perinuclear Region; and (**c**) Cytoplasm. Spectra are vertically offset for better visibility.

The effect of Bortezomib in the biochemical milieu of cells was further determined by analyzing mean Raman spectra extracted from each of the clusters ascribed to an intracellular region. As shown in Figure 3a, spectra of the nuclear region is dominated by nucleic acid features indicated by bands at 720, 746, 786, 826, 895, 1093, 1320 and 1424 cm^−1^. In addition to morphological differences, spectra from nuclear region of treated cells have spectral differences as compare to untreated cells. Decrease in nucleic acid concentrations in Bortezomib-treated cells is shown by the reduced intensity of 720, 746 and 786 cm^−1^ bands assigned to ring breathing modes of nitrogen bases. This can be due to the DNA damage and DNA-repair pathway inhibition in Bortezomib-treated cells. Minor differences in protein conformation shown by the spectral differences around 947 cm^−1^ (proline/valine), 985 cm^−1^ (c-c stretching in B-sheet) and amide III region were also observed. Typically, lymphocytes consist of a large nucleus surrounded by scantly distributed cytoplasm. Some lymphocytes have a clear perinuclear zone (or halo) around the nucleus or could have a small clear zone to one side of the nucleus. Spectra extracted from this region (yellow), shown in Figure 3b, suggest that treated and untreated cells have similar profiles consisting of both nucleic acid and protein related bands. Minor differences observed in the amide III region could be ascribed to the drug effect on proteins in this region. Earlier observations corroborate the decrease in the nucleic acid concentrations indicated by bands at 786 and 1340 cm^−1^. Spectra extracted from the cluster attributed to the thin layer of cytoplasm demonstrate visible differences between treated and untreated cell. Major changes in the concentrations of several amino acids (proline/hydorxyproline (820 cm^−1^), tyrosine (856 cm^−1^), tryptophan (880 and 1208 cm^−1^), and c-c backbone of proteins (940 cm^−1^)), phosphorylated proteins, and nucleic acids (970 cm^−1^) were observed. Broadening of amide I and III regions in Bortezomib-treated cells suggests changes in the secondary structure of proteins. Previous cytochemical studies have shown that glycogen content in the cytoplasm can be used as a marker for proliferating lymphocytes [28]. Differences around the 1048 cm^−1^ band assigned to glycogen could be due to effects of Bortezomib. Pully et al., performed time-lapse Raman imaging coupled with cluster imaging on peripheral lymphocytes [29] and demonstrated that the cytoplasm has contributions from lipid, proteins, and carotenoids. The presence of carotenoids in the lymphocytes could be due to their important role in the immune response as quenchers of singlet oxygen species. Observed differences at 1154 and 1524 cm^−1^ bands, corresponding to carotenoids, corroborate with these findings [29]. All the band assignments were performed on the basis of the previously reported literature [30]. Overall, cluster imaging and spectral profiling of individual cellular components suggest that drug-induced effects on cellular pathways can be identified in a label-free and quantitative manner with our current Raman setup. Further follow-up with additional time points could provide information about temporal changes. We suspect that there could be some minor changes attributed to changes in ambient temperature, absence of nutrients in the media, and lack of maintaining pH levels over the course of the experiment. Our future research will focus on integrating a mini-bioincubator into the Raman setup to maintain physiological parameters and improve the cell viability. 

### 3.3. Study on Group of Cells

**Figure 4:**
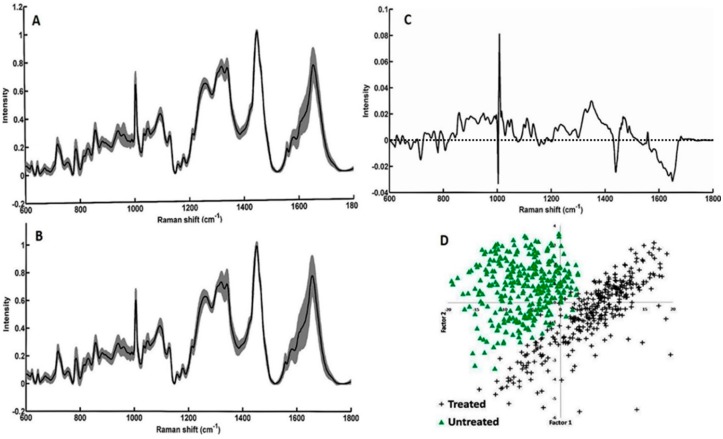
Mean baseline corrected spectrum along with standard deviation (shaded region) acquired from group of cells: (**A**) Untreated; (**B**) Treated; (**C**) difference spectrum (Untreated − treated); and (**D**) PCA scatter plot.

**Figure 5:**
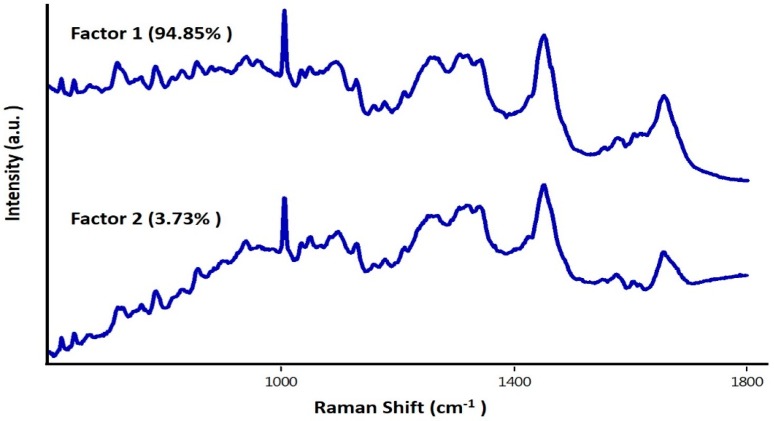
Loading plots of factors used for PCA.

To further evaluate the feasibility of our setup to acquiring data from large number of cells in a short time period, spectra from ~400 cells were obtained. The average spectra and standard deviations are shown in Figure 4. Bands related to both nucleic acids and proteins were observed. Visible differences among both groups could be linked to the Bortezomib-induced changes of characteristic peaks corresponding to the symmetric and asymmetric stretching of nucleic acids (600–800 cm^−1^), backbone geometry, and phosphate ion interaction (800–1200 cm^−1^), suggesting structural degradation of the nuclear material. A minor blue shift in the phenyalanine band (1003 cm^−1^) was also observed in the Bortezomib-treated cells. These differences were further enhanced for visualization by computing the difference spectra (untreated − treated). In this case, all positive bands belonged to the untreated group of cells and all the negative bands belonged to treated cells. Similar to the changes previously observed in the nuclear component of individual cell, positive bands around 783, 788, 804, 1340, and 1490 cm^−1^ can be predominantly observed in the untreated group (Figure 4C). Other positive bands at 1053 cm^−1^, 1123 cm^−1^ (C-N stretching of proteins), amide III, 1460 cm^−1^ (lipids) and 1558 cm^−1^ (tryptophan) suggest high protein concentration in the untreated cells compared to the treated cells [30]. Strong bands around the phenylalanine region could be due to the minor blue shifts in treated cells reported above. Predominant negative bands around 1440 cm^−1^ (CH_2_ deformation) and 1660 cm^−1^ (amide I) could be due to changes in the secondary structure of proteins from the Bortezomib treatment. The spectral profiles obtained from group of cells support earlier observations on individual cells. To verify if these differences can be utilized for differentiating between treated and untreated cells, PCA was performed. As shown in Figure 4D, two separate clusters of “treated” and “untreated” cells were obtained under PCA. Spread in the cluster of “treated” group could be due to the differential effect of Bortezomib during cell cycle division. As Bortezomib induces apoptosis, it will have varying effects on each cell depending on the cell cycle phase. Loading plots of factors, along with percentage contributions used for classification, are shown in Figure 5. Majority of differences responsible for discrimination seems to originate from factor 1. Corroborating spectral variability indicated by difference spectra (Figure 4C), loading plots also suggest major differences in nuclear and protein content of the cells. Shifting in phenylalanine band also contributes to the classification. Major differences between factor 1 and 2 is in the amide I band. These findings support earlier observation regarding mode of action of the drug. As demonstrated on single cell, the drug works by influencing the nuclear composition, disrupting the intracellular demarcation and ultimately leading them towards apoptosis. Overall, findings support the utility of the current set-up in identifying earliest changes in group of cells, suggesting its applicability for label-free monitoring drug response in a fast and sensitive manner.

Real time analysis of the interaction between drugs and cells to monitor uptake and determine cell fate continues to be a growing area of research interest. As the focus of pharmaceutical research shifts towards the development of nano-drug carriers and personalized medicine, novel tools for monitoring therapeutic responses in real-time are critical. Optical microscopy has been used routinely for studying cell morphology and growth rate. However, current methods are limited and qualitative since they do not provide molecular or chemical information [31]. Electron microscopy and atomic force microscopy can provide high resolution up to sub-cellular level but have limited applicability due to its destructive nature and only morphological changes can be observed. Confocal fluorescence microscopy is the most common technique for cell visualization. Antibody-coupled fluorophores specific to different cell organelles can provide valuable information. However, this technique is limited to the number of fluorophores that can be used due to the limited range of excitation wavelengths that can be used. Confocal fluorescence microscopy is also susceptible to photo-bleaching effects making long term monitoring unfeasible [31]. Moreover, it is not applicable to human patients due to the staining process. Optical approaches especially Raman spectroscopy provide high chemical sensitivity in a non-destructive and label-free manner. This technique could be potentially applicable for in vivo drug monitoring which is the critical process for personalized treatment of patients. In this report, we have presented a high-resolution high-speed confocal Raman system capable of imaging cells in vitro, and has great potential as a viable method for studying live cell dynamics. We have successfully demonstrated the effects of Bortezomib on intracellular components of multiple myeloma cells with Raman microscopy. Disturbance in the nuclear components coupled with the morphological rearrangements are the distinguishing features between treated and untreated cells. We also showed that high quality spectra from large number of cells can be acquired in a short time period. This could lead the way for developing a new in vivo drug monitoring device for personalized treatment. Dosage of drug can be adjusted by monitoring cellular response in real-time. Using the system reported here, high-speed confocal Raman microscopy could become a powerful tool for the simultaneous real-time monitoring of the cellular and chemical changes of cells under various treatments or interventions.
